# Tubo-ovarian abscess as an initial presentation of varicella in 12 years old child; a case report

**DOI:** 10.1093/omcr/omaf224

**Published:** 2025-12-27

**Authors:** Nabeeha N Akram, Shaymaa K Abdulqader, Wassan N Mohammed, Qays A Hassan

**Affiliations:** Department of Pediatrics, Mustansiriyah University, Al-Sakhra Intersection, Palestine Street, Baghdad 10064, Baghdad, Iraq; Department of Radiology Al-Kindy College of Medicine, University of Baghdad, Al-Nahdha square, P.O Box 47188, Al-rusafa District, Baghdad, Iraq; Department of Obstetrics and Gynecology, Mustansiriyah University, Palastine Street, Baghdad 10064, Iraq; Department of Radiology Al-Kindy College of Medicine, University of Baghdad, Al-Nahdha square, P.O Box 47188, Al-rusafa District, Baghdad, Iraq

**Keywords:** infectious diseases and tropical medicine, radiology, emergency medicine

## Abstract

Varicella (chickenpox) caused by varicella zoster virus is usually diagnosed clinically based on the typical presentation of fever and vesicular rash in immunocompetent children. However, atypical sites of the rash or non-cutaneous suppurative complications can cause a diagnostic dilemma. We report a tubo-ovarian abscess as an initial presentation of chickenpox in a 12-year-old female, a novel and unrecognized presentation of varicella in childhood. Unlike previously reported cases with varicella associated abscess that treated by intravenous antibiotics and surgical drainage, the current patient did not receive any medical or surgical therapy and spontaneous resolution of the abscess radiologically confirmed on serial follow up with imaging.

## Introduction

Varicella, commonly referred to as chickenpox, is a common self-limiting viral disease caused by Varicella Zoster Virus (VZV). It affects all age groups but is mostly acquired in childhood. Typically presented as fever and vesicular rash mostly involves the trunk, face, and scalp, with atypical rash involving inflamed skin or in sun-exposed areas is increasingly reported [1]. Although it runs a benign course in most immunocompetent children, severe, life-threatening complications are a possibility. These are divided into cutaneous or extracutaneous (systemic) complications. The cutaneous complications are varied, encompassing secondary bacterial skin infections, necrotizing fasciitis, varicella gangrenosa, post-varicella scarring, cutaneous vasculitis, Stevens–Johnson syndrome and toxic epidermal necrolysis [2].

Although rare, abscesses have been reported as an initial manifestation of varicella in children in different sites, including retropharyngeal, lung, and bone [3, 4]. When an abscess is apparent and the rash is already recognized, the management will be at ease. The dilemma is when an abscess occurs in deep structures, and it precedes the onset of a varicella rash. This atypical presentation can lead to diagnostic uncertainty and may subject the child to unnecessary diagnostic and interventional procedures [5].

Timely diagnosis and implementation of appropriate therapeutic measures are critical. The management of abscesses associated with varicella relies on surgical drainage with intravenous antibiotics [1]. A meticulous review of the current medical literature revealed no cases of tubo-ovarian abscess directly associated with varicella zoster virus. The patient in the present case represents a novel etiology of pelvic abscess as chickenpox, which has not previously been reported to present as pelvic abscess. In addition, the spontaneous resolution of this abscess without any medical and surgical intervention is worth reporting.

## Case report

A previously healthy 12-year-old female presented with a 4-day history of flu-like symptoms, generalized malaise, and low-grade fever, accompanied by progressively worsening pain localized to the left iliac fossa. The abdominal pain increased in severity, eventually interfering with ambulation and sleep. The clinical course was further complicated by multiple episodes of non-projectile vomiting, prompting hospital admission. Upon presentation, the child appeared acutely ill. Vital signs included an axillary temperature of 37.5°C, pulse rate of 100 beats per minute, respiratory rate of 25 cycles per minute, and oxygen saturation of 96% on room air. Her anthropometric measurements revealed a height of 148 cm and a body weight of 40 kg, both falling below the 50th percentile for age. No rash or other cutaneous manifestations all over the body. Laboratory tests were normal apart from leukocytosis with WBC = 16 × 10^9^ cells/l.

**Figure 1:**
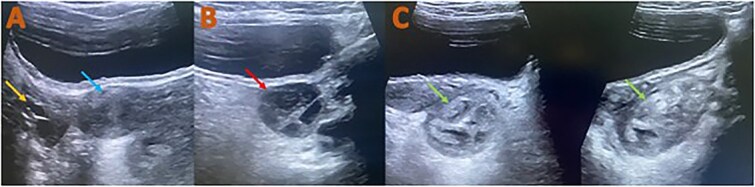
Ultrasound of the pelvis: A) shows the normal size and texture of the right ovary and normal uterus. B) Shows normal size and texture of the left ovary. C) shows left adnexal well defined echogenic mass lesion showing internal heterogenous texture measured (38 × 40 × 50mm = 40 cc) and avascular on color doppler US.

Chest examination was unremarkable, but on abdominal examination, left iliac fossa tenderness with focal guarding and positive psoas sign as an inability to straighten her left thigh, but no palpable organomegaly or abdominal mass was detected. On pelvic ultrasound, a well-defined Left adnexal heterogeneous echogenic mass lesion with internal cystic areas located between the uterus and Left ovary surrounded by echogenic fat (Fig. 1).

**Figure 2:**
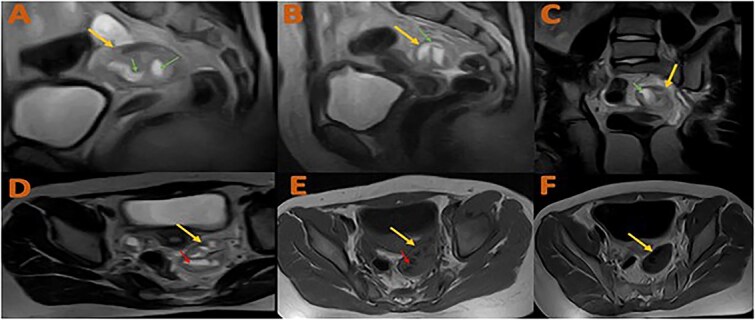
Contrast-enhanced MRI of the pelvis. (A and B) sagittal T2WI. (C) Coronal T2WI; (D) axial T2WI; (E and F) axial T1 TWI pre- and post-contrast administration. Shows well-defined heterogenous mass involving the left adnexa between the uterus and left ovary ( arrows), which contains dilated fluid-filled T2 hyperintense fallopian tube with multiple fluid levels (small arrows). The mass shows no enhancement post-contrast administration.

An ovarian tumor was suspected, prompting further evaluation by pelvic MRI and tumor markers. Pelvic MRI revealed a well-defined left adnexal mass lesion with low SI on T1WI, high SI on T2WI, with an internal fluid-filled tubular structure exhibiting peripheral enhancement and surrounded by edematous enhancing soft tissues (Fig. 2). The tumor markers were all negatives: ca-125 = 12.5 U/ml (reference value: less than 35 U/ml), B-HCG = 5 mIU/ml (normal non pregnant is less than 5 mIU/ml), AFP =1.7 ng/ml (normal value less than 9.5 ng/ml). An isolated tubal torsion was suspected, and the patients scheduled for surgery. Next day the patients started to have multiple skin lesions in the form of fluid-filled blisters scattered over the face, abdomen and back which is itchy, so the surgery postponed two days, in this time the rash was increased in number and severity, but the Lt iliac fossa pains and fever were resolved. Based on characteristic rash the patient diagnosed as chickenpox.

**Figure 3:**
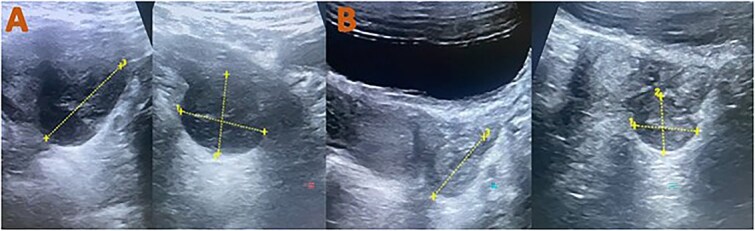
Follow-up pelvis ultrasound exams: (A) after 2 weeks the previously visualized left adnexal mass became more circumscribed and showed internal cystic texture measured (30 × 28 × 37 mm = 16 cc volume). (B) 6 weeks later: The mass had decreased in size and developed a heterogeneous echogenic texture, measuring 13 × 16 × 24 mm (3 cc volume).

On repeated pelvic US, the mass slightly increased in volume (from 27 cc to 40 cc), despite clinical improvement of the child’s general status (Fig. 3A). The patient subsequently discharged from the hospital and kept under follow up with serial pelvic ultrasonography. Ten days post discharge, follow up US showed the mass displayed cystic changes and markedly decreased in volume to 13 cc. At a six-week follow-up, pelvic ultrasound indicates that the mass is markedly decreased in size, measuring 3 cc in volume (Fig. 3B).

## Discussion

Pelvic abscess is uncommonly encountered in premenarchal girls, especially in sexually inactive adolescents as it frequently arises as complication of pelvic inflammatory bowel disease [6]. However, it has been rarely reported as a first manifestation of other diseases including inflammatory bowel diseases [7]. Regardless of etiology, abdominal pain is the predominant presenting symptom with various other presentations involving fever, nausea, vomiting, dysuria, and diarrhea [6]. To the best of our knowledge, no cases of tubo-ovarian abscess in association with varicella infection in pediatric patients have been reported previously.

Although chickenpox theoretically can result in abscess anywhere in the body resulting from skin barrier disruption and transient immunosuppression with subsequent secondary bacterial infection but skin abscess where the predominantly reported site [4]. Previously reported extracutaneous abscess in chickenpox as summarized, in Table 1. There is an obvious female predominance in abscess linked to varicella with age range (3–60) months which is much younger than age reported in current case. All patients previously reported with abscess linked to varicella in pediatric patients were treated by antibiotics with or without surgical drainage (Table 1). However, the current case did not receive any antibiotics and was kept on watchful waiting with serial radiological follow up which eventually revealed a spontaneous resolution of the abscess without medical or surgical interventions.

**Table 1:** Summary of reported varicella cases in children presented as extracutaneous abscess.

Sites	Presentation	Age (months)	Gender	Medical Treatment	Surgical	Refs
Retropharyngeal abscess	Neck swelling, decreased oral intake and urine output.	19	Female	Intravenous antibiotics	Incision and drainage	[8]
Retropharyngeal abscess	Persistence of fever, cough, odynophagia, dysphagia	48	Female	Intravenous antibiotics	Surgical drainage.	[9]
Neck abscess	Bilateral painful neck swelling, fever	17	Male	Intravenous double antibiotics	Surgical drainage	[10]
Lung abscess	high fever, cough, and respiratory distress.	3	Female	Intravenous double antibiotics and acyclovir	Noninvasive ventilation	[4]
Intraperitoneal abscess with perforated appendicitis	diarrhea, vomiting, and malaise. guarding and severe abdominal pain in the right lower quadrant	60	Female	Intravenous double antibiotics and acyclovir	Appendectomy, Abscess drained using ultrasound guided technique	[5]
Subperiosteal in femur	Febrile convulsion. Leg pain	31	Female	Triple broad-spectrum intravenous antibiotics	Urgent aspiration and repeated washout with subsequent surgical drainage.	[3]

The present case highlights a novel presentation of chickenpox with tubo-ovarian abscess in children. It underscores the need for pediatricians to consider varicella in the differential diagnosis of acute abdomen in children, particularly during outbreaks, and to remain alert for atypical and severe bacterial complications during the viremic phase of VZV.

## Notes

The parents of the patient gave signed informed consent for publication of the case with all related pictures and information.

Data access: The data that support the findings of this report available from the corresponding author upon reasonable individual request.

The paper has not been previously published, nor is it concurrently submitted to any other journal.

The final version of the manuscript has been read and approved by all authors.

## References

[1] Mazzara C, Milani GP, Lava SAG. et al. Atypical primary varicella rash: systematic literature review. Acta Paediatr 2022;111:935–9. 10.1111/apa.16300 35178772 PMC9306993

[2] Tabasizadeh H, Mahmoudi S, Khodabandeh M. et al. Severe skin complications of varicella in previously healthy children in Iran: emerging concern. BMC Infect Dis 2025;25:402. 10.1186/s12879-025-10794-w 40128649 PMC11934440

[3] Lim JBT, Huntley JS. Musculoskeletal sequelae of varicella-zoster infection: two case reports. Scott Med J 2012;57:121. 10.1258/smj.2011.012010 22555234

[4] Aygun D, Aygun F, Kılınc AA. et al. Lung abscess from Staphylococcus aureus after varicella infection in a 3-month-old infant. J Infect Public Health 2017;10:129–32. 10.1016/j.jiph.2016.05.013 27349422

[5] Smedegaard LM, Christiansen CB, Melchior LC. et al. Appendicitis caused by primary varicella zoster virus infection in a child with DiGeorge syndrome. Case Rep Pediatr 2017;2017:6708046. 10.1155/2017/6708046 28900551 PMC5576433

[6] Anant M, Sinha K. Pelvic abscess in a sexually inactive teenage girl: a case report and review of literature. Int J Reprod Contracept Obstet Gynecol 2021;10:1713–6. 10.18203/2320-1770.ijrcog20211165

[7] Hartmann KA, Lerand SJ, Jay MS. Tubo-ovarian abscess in virginal adolescents: exposure of the underlying Etiology. J Pediatr Adolesc Gynecol 2009;22:e13–6. 10.1016/j.jpag.2008.03.006 19539189

[8] Clark CM, Huntley C, Carr MM. Varicella infection complicated by group a Beta-Hemolytic streptococcal retropharyngeal abscess. Case Rep Otolaryngol 2016;2016:9298143. 10.1155/2016/9298143 27651967 PMC5019878

[9] Menéndez del Castro M, Coca-Pelaz A, Menéndez S. et al. Retropharyngeal abscess and mediastinitis as an uncommon complication of varicella infection. Int J Pediatr Otorhinolaryngol 2020;132:109904. 10.1016/j.ijporl.2020.109904 32018164

[10] Palanimuthu D, Yong E, Ramachandran K. et al. Neck abscess as a rare sequela of Pediatric varicella-zoster infection: a case report. Cureus 2025;17:e77639. 10.7759/cureus.77639 39968434 PMC11833142

